# Now for New York. September, 1899

**Published:** 1899-06

**Authors:** 


### NOW FOR NEW YORK—SEPTEMBER, 1899.

ALMOST every feature of the coming meeting is now outlined, and the programme offers something of interest for every member of the Association, for every devotee of the profession. The routine practitioner will have spread for his delectation a round of good things scarcely ever before equalled. The specialist in our several lines of sanitary police work will find himself well provided for in the consideration of subjects of paramount interest to the whole world. Those of our members specially fitted to present forcefully these various aspects of our work have been well chosen and are peculiarly well equipped, while everyone has something to contribute to more fully cover every feature of these subjects. The time is auspicious for a grand meeting; prosperity again reigns over our calling. The place of meeting has every attraction and is fittingly held in a centre that played so important a part in the early history of our Association. The Secretary has been untiring in his efforts to make the meeting a success. The local committee of arrangements are fully responsive to every demand made upon them, and are keen in their desire to afford everyone a generous welcome, a good time in general, a special feast of good things, and there is not a good reason left for any absentees, and the profession in New York should turn out *en masse.*

---

The JOURNAL welcomes to the support of the veterinary schools of our land the State and sectional alumni bodies forming over our land, to sustain the parent institutions of which they are graduates. McGill's Veterinary Department has a strong New England alumni body to sustain her proud record and encourage by voice and action her splendid work. The American Veterinary College now adds a New England alumni body of her graduates to second and foster all her plans and purposes and to maintain the honorable career the parent body has maintained. The Ontario Veterinary College has also in the Bay State an alumni body to give assistance to their college parent and aid her in her future efforts to sustain and advance the long career of constant service she has maintained in the field of veterinary education. More of these organizations should be formed and a keener interest retained in all the colleges by their outgoing representatives. It adds pleasure to the giver and strength to the recipient of all such tokens of recognition. Let the good work go on.

